# An Interesting Case Report of Myasthenia Gravis Exacerbation Induced by Durvalumab

**DOI:** 10.7759/cureus.26985

**Published:** 2022-07-18

**Authors:** Oluseyi Abidoye, Nathan Kim, Jason Fombi

**Affiliations:** 1 Internal Medicine, Northeast Georgia Medical Center, Gainesville, USA

**Keywords:** non-small-cell lung carcinoma, cancer immunotherapy, adverse effects, myasthenia gravis exacerbation, immune check-point inhibitor

## Abstract

Immune checkpoint inhibitors are novel therapy for a wide range of malignancies. They have been associated with numerous side effects resulting in pulmonary, dermatological, gastrointestinal, and neurological complications. There are few reported cases of myasthenia gravis exacerbation from immune checkpoint inhibitors. We present a case of an 82-year-old woman with a history of myasthenia gravis in remission and non-small cell lung cancer who presented with diplopia, dyspnea, and generalized weakness after three cycles of durvalumab. She was diagnosed with a myasthenic crisis and was treated with high-dose steroids and plasmapheresis.

## Introduction

Immune checkpoint inhibitors (ICI) are new treatment therapies used for the treatment of various malignancies. Durvalumab is a Food and Drug Administration (FDA)-approved monoclonal antibody that prevents the interaction of programmed cell death ligand 1 (PD-L1) with the PD-1 (CD279). It is a novel therapy that has been approved for the treatment of urothelial carcinoma, non-small cell lung cancer, and extensive small cell lung cancer. Regardless of this breakthrough therapy, immune checkpoint inhibitors have been associated with immune-related adverse effects (irAE). The most common irAE associated with durvalumab include pneumonitis (2.4%), colitis (2%), hepatitis (2.8%), dermatological reactions (1.8%), and endocrinopathies such as thyroid dysfunctions (0.5- 8.3%), adrenal insufficiency (0.5%), diabetes mellitus and hypophysitis (0.1%) [1]. Durvalumab-induced myasthenia gravis (MG) is a rare complication, with few reported cases in the current literature [2]. We report a case of a patient who developed myasthenia gravis crisis after receiving durvalumab.

## Case presentation

An 82-year-old Caucasian female with a history of hyperlipidemia, hypertension, hypertrophic nonobstructive cardiomyopathy, sick sinus syndrome secondary, monoclonal gammopathy of unknown significance (MGUS), ductal carcinoma in situ (DCIS) of right breast diagnosed in 2012 was treated with lumpectomy, radiation therapy and hormonal therapy. She also had a history of myasthenia gravis secondary to thymoma treated with thymectomy in remission since 2020 and was first diagnosed with right middle lobe squamous cell carcinoma of the lung in May 2021. She underwent computed tomography (CT) of the abdomen and pelvis revealing a 2.9cm x 1.5cm soft-tissue density within the right middle lobe. She underwent CT guided core biopsy of the right lung. Pathology revealed non-small cell carcinoma consistent with squamous cell carcinoma. Tumor cells were positive for P40, thyroid transcription factor 1 (TTF-1), and negative GATA-3, B72.3. The PDL-1 expression tumor proportion score was high at 65%. Subsequent positron emission tomography (PET) revealed several fluorodeoxyglucose (FDG)-avid mediastinal, subcarinal and right hilar lymph nodes suggestive of metastasis. She was diagnosed with T1cN2, MO stage IIIA.

The patient was started on combined chemotherapy of taxol and carboplatin weekly along with radiation for six to seven weeks which she completed in August 2021. She was initiated on adjuvant durvalumab for maintenance therapy and received three cycles (10mg/kg) with the last cycle in October 2021. She presented after three days of receiving the third cycle of immunotherapy with complaints of a four-day history of shortness of breath, double vision, and generalized weakness. Physical examination was significant for ptosis of the left eyelid with limitation in abduction of both eyes and muscle weakness with muscle strength of 3/5 in both upper extremities. A CT of the brain was negative. Neurology was consulted, a diagnosis for myasthenic crisis was made based on symptoms and decreased negative inspiratory force. Patient was started on pyridostigmine 60mg twice daily, prednisone at 1mg/kg/day and underwent plasmapheresis completing only two out of five rounds. On hospital day five, the patient refused any further plasmapheresis treatment requesting to be transitioned to comfort measures. After multiple goal of care discussions, patient was transitioned to comfort measures. The patient died on the sixth day of admission. 

## Discussion

This case report highlights a rare case of exacerbation of myasthenia gravis because of PD-L1 inhibition with durvalumab. Myasthenia gravis is a paraneoplastic syndrome associated with thymoma and lung carcinoma [3]. There have been several reported cases of MG induced by durvalumab [2]. Myasthenia gravis induced by ICI is a rare irAE with an incidence of less than 1% [1]. It has been reported that MG related to ICI often occurs between two and six weeks after initiation. Even though our patient was not on any maintenance therapy for MG, we concluded the likely cause for her exacerbation was related to the initiation of durvalumab. A recent retrospective study involving immunotherapy with anti-PD1 nivolumab showed that 66% of patients developed severe irAE which included myasthenic crisis. [4] It was reported that patients who developed MG had an early onset during the treatment course with rapid progression, similar to our patient's clinical course. A recent systematic review and meta-analysis of the association of ICIs with neurologic adverse events suggested that the overall risk of neurologic adverse events (NAEs), peripheral neuropathy, and dysgeusia was lower with the use of ICIs when compared with chemotherapy, but higher when compared with placebo [5]. Despite the rarity of adverse events, prompt identification is advised to provide respiratory support either through noninvasive positive pressure ventilation or mechanical ventilation to prevent patients from experiencing acute respiratory failure. In patients with mild symptoms, acetylcholinesterase inhibitors such as pyridostigmine are recommended. However, in patients with severe presentations, immunosuppressive agents such as intravenous methylprednisolone are required. Studies have reported better outcomes with plasma exchange or intravenous immunoglobulin (IVIG) as first-line therapy compared to steroids alone [6]. Overall, the majority of irAEs are treated with high-dose steroids; immunosuppressants such as mycophenolate, infliximab, alemtuzumab, abatacept; and in some cases IVIG [7].

## Conclusions

Immune checkpoint inhibitors have been reported to have lesser side effects and better tolerability than chemotherapy, but they are known to have side effects that can be detrimental and potentially lead to life-threatening end-organ damage. It is crucial that physicians who treat patients with ICI are aware of the potential side effects and complications associated with ICI and ensure they can provide prompt care and management for patients with these complications. 
